# The Role of Reactive Oxygen Species and Nitric Oxide in the Inhibition of *Trichophyton rubrum* Growth by HaCaT Cells

**DOI:** 10.1155/2020/8548619

**Published:** 2020-02-12

**Authors:** Meiling Huang, Hao Huang, Wenyi Lv, Hanyue Xiao, Ye Gao, Hongfeng Tang

**Affiliations:** ^1^Department of Dermatology, Huzhou Central Hospital, 198 Hongqi Rd, Huzhou, 313000 Zhejiang, China; ^2^Department of Dermatology, Shunde Hospital, Southern Medical University (The First People's Hospital of Shunde Foshan), No. 1 Jiazi Road, Lunjiao, Shunde District, Foshan City, Guangdong Province, China 528308

## Abstract

*Trichophyton rubrum* (*T. rubrum*) is one of the most important agents of dermatophyte infection in humans. The aim of this experiment was to evaluate the effect of HaCaT cells on *T. rubrum*, investigate the responsible mechanism of action, and explore the role of reactive oxygen species (ROS) and nitric oxide (NO) in the inhibition of *T. rubrum* growth by HaCaT cells. The viability of fungi treated with HaCaT cells alone and with HaCaT cells combined with pretreatment with the NADPH oxidase inhibitor (DPI) or the nitric oxide synthase (NOS) inhibitor L-NMMA was determined by enumerating the colony-forming units. NOS, ROS, and NO levels were quantified using fluorescent probes. The levels of the NOS inhibitor asymmetric dimethylarginine (ADMA) were determined by enzyme-linked immunosorbent assay (ELISA). Micromorphology was observed using scanning electron microscopy (SEM) and transmission electron microscopy (TEM). In addition, fungal keratinase activity was assessed by measuring dye release from keratin azure. In vitro fungal viability, keratinase activity, and ADMA content decreased after HaCaT cell intervention, whereas the levels of ROS, NO, and NOS increased. The micromorphology was abnormal. Fungi pretreated with DPI and L-NMMA exhibited opposite effects. HaCaT cells inhibited the growth and pathogenicity of *T. rubrum* in vitro. A suggested mechanism is that ROS and NO play an important role in the inhibition of *T. rubrum* growth by HaCaT cells.

## 1. Introduction

*Trichophyton rubrum* (*T. rubrum*) is a dermatophyte responsible for the majority of fungal infections worldwide [1] and accounts for as many as 69.5% of dermatophytoses in humans [2]. Although dermatomycoses are frequently limited to superficial keratinized tissues such as nails, skin, and hair, fungi may invade the dermis and subcutaneous tissue, especially when precipitated by trauma, immunosuppression, or the use of topical corticosteroids [3].

In fungal infectious diseases, Langerhans cells, neutrophils, and macrophages in the stratum corneum appear to be important in the defense against fungi, such as *Malassezia* spp. and *T. rubrum* [4]. In addition, the exposure of keratinocytes to *T. rubrum* leads to induced expression of antimicrobial peptides (AMPs), and increased levels of AMPs may help the host control the growth and spread of *T. rubrum* and most likely other dermatophytes [5]. Therefore, we hypothesized that keratinocytes may perform a defensive function against *T. rubrum*. However, the underlying mechanism is not entirely clear.

*T. rubrum* is highly adapted to human immunity. Fungi are extracellular pathogens, and the fungal cell wall components are highly conserved and are thus termed pathogen-associated molecular patterns (PAMPs), which have an important role in pathogen recognition by the host immune system [6]. Keratinocytes, the first line of defense against infection, can directly recognize conserved PAMPs and initiate the immune response via their pattern recognition receptors (PRRs) [7], including Toll-like receptors (TLRs) and c-type lectin receptors (CLRs) [8, 9]. Then, keratinocytes secrete endogenous antimicrobial cytokines and actively mediate skin immunity [10, 11]. Although emerging evidence has proven that keratinocytes play a crucial role as participants in antifungal defense, the mechanism mediating the fungal response to keratinocytes is not understood.

Many studies have demonstrated that external stimuli, such as phototherapy or host cell defense, trigger the production of reactive oxygen species (ROS) and nitric oxide (NO) in fungal cells, which are molecules that appear to be critical for killing *T. rubrum* [12, 13]. The fate of the fungus during this exposure to external stress is dependent on free radicals, especially ROS and NO [14]. Common ROS include singlet oxygen (^1^O_2_^−^), superoxide radicals (O_2_^·-^), hydroxyl radicals (OH^·^), and hydrogen peroxide (H_2_O_2_) [15]. ROS can cause widespread cascades of oxidative damage, such as lipid peroxidation, protein carbonylation, and DNA damage through strand breaks and DNA-protein crosslinking [16]. The consequence is damage to the organelles, cell membrane, and cell walls, ultimately resulting in cell death.

NO is a free radical that reacts with oxygen to form oxides of nitrogen [17]. NO, a gaseous immunomodulator with multimechanistic antimicrobial activity, is formed by the oxidation of L-arginine by nitric oxide synthase (NOS) in eukaryotic cells [18]. NO has been reported to have antimicrobial activity against bacteria, fungi, and viruses both in vitro and in vivo [19–21]. Its antifungal activities have been reported to delay mycelial growth and conidial germination [22, 23]. In addition, numerous studies have shown that NO exerts direct fungistatic activity via DNA damage, lipid peroxidation, and enzyme inactivation [24]. Furthermore, ROS and NO react with each other as well, resulting in the generation of peroxynitrite [25]. The ROS and NO comprise a key axis of the host-pathogen interaction.

In this study, we aimed to investigate the mechanism stimulated when *T. rubrum* attacks its host and sought to determine whether ROS and NO play a role in inhibiting the growth of *T. rubrum* by HaCaT cells, a human keratinocyte cell line.

## 2. Methods

### 2.1. Fungus Source

The *T. rubrum* strain ATCC4438, obtained from the American Type Culture Collection (ATCC), was cultured on potato dextrose agar (PDA) plates for 7 days at 26°C.

### 2.2. Keratinocytes

The immortalized human keratinocyte cell line HaCaT was purchased from the China Center for Type Culture Collection (CCTCC). The cells were cultured in high-glucose Dulbecco's Modified Eagle's Medium (Gibco, America) supplemented with 10% fetal bovine serum at 37°C in a humidified atmosphere containing 5% CO_2_.

### 2.3. Detection of Fungal Activity

At a confluence of 80%-90%, HaCaT cells were exposed to high-glucose Dulbecco's Modified Eagle's Medium (Gibco, America) containing a suspension of *T. rubrum* in a humidified atmosphere with 5% CO_2_. Because the growth environment with or without carbon dioxide has a large effect on the cells but has little effect on the fungi, the experiments were performed in a 5% carbon dioxide incubator when the cells were cocultured with the fungus. For the time-response assay, HaCaT cells were treated with the *T. rubrum* suspension for 12 h and 24 h. For the dose-response assay, the ratios of HaCaT cells to *T. rubrum* suspension were 1 : 1, 2 : 1, and 4 : 1 (1 : 1 means 5∗10^6^ to both cells and fungus, 2 : 1 means 5∗10^6^ cells to 2.5∗10^6^ fungus, and 4 : 1 means 5∗10^6^ cells to 1.25∗10^6^ fungus). A *T. rubrum* suspension was used as the control. To analyze the role of ROS and NO in HaCaT cell intervention, we pretreated the fungus with DPI (a Nox inhibitor) or L-NMMA (a NOS inhibitor) for 2 h before coculture with HaCaT cells, according to the methods of Huang et al. [26–28]. We assessed fungal viability by enumerating the colony-forming units. As the fungal activity worsened, the fungal colonies in the plate decreased. Finally, the growth inhibition rates at each time point and each dose were calculated.

### 2.4. Determination of ROS Levels

Based on the results of the above tests, a ratio of HaCaT cells to *T. rubrum* suspension of 4 : 1 and a coculture time of 24 h were used.

We divided the cultures grown in the liquid medium into a *T. rubrum* group, a *T. rubrum*+HaCaT cell group, and a *T. rubrum*+DPI+HaCaT cell group. We pretreated the fungus with DPI for 2 h before coculture with HaCaT cells.

The total intracellular ROS level was evaluated using 10 mM 2′,7′-dichlorofluorescein diacetate (DCFH-DA) (Beyotime Biotechnology, Haimen, China), a nonfluorescent probe. DCFH-DA itself has no fluorescence and can freely cross the cell membrane. After entering the cell, it can be hydrolyzed by the esterase in the cell to generate DCFH. DCFH does not penetrate the cell membrane, which makes it easy to load probes into cells. ROS in cells can oxidize nonfluorescent DCFH to generate fluorescent DCF. Detection of DCF fluorescence reflects the level of ROS in the cell. The *T. rubrum* suspension was incubated with DCFH-DA at 28°C for 20 min in a CO_2_ incubator and then washed three times with PBS. The fluorescence signal intensity was analyzed using a flow cytometer (BD FACSCanto II) at an excitation wavelength of 488 nm and an emission wavelength of 525 nm.

### 2.5. Determination of NO Levels

We divided the cultures grown in liquid medium into a *T. rubrum* group, a *T. rubrum*+HaCaT cell group, and a *T. rubrum*+L-NMMA+HaCaT cell group. We pretreated the fungus with L-NMMA for 2 h before coculture with HaCaT cells. A *T. rubrum* suspension of each group was incubated with 5 *μ*mol/l 3-amino,4-aminomethyl-2′,7′-difluorescein diacetate (DAF-FM DA) (Beyotime Biotechnology, Haimen, China), a fluorescent probe, at 37°C for 20 min in a CO_2_ incubator and washed in PBS three times. DAF-FM DA can pass through the cell membrane. After entering the cell, it can be catalyzed by intracellular esterase to form DAF-FM, which cannot pass through the cell membrane. DAF-FM itself has only very weak fluorescence but can generate strong fluorescence after reacting with nitric oxide; thus, the degree of NO production can be detected. The fluorescence signal intensity was analyzed using a flow cytometer (BD FACSCanto II) at an excitation wavelength of 495 nm and an emission wavelength of 515 nm. Based on the fluorescence signal intensity, we could detect the level of NO in *T. rubrum*.

### 2.6. Determination of NOS Level Activity

We used a fluorescent probe method to detect the level of NOS activity. This method uses a nitric oxide fluorescent probe (DAF-FM DA) to detect the amount of nitric oxide that can be produced by NOS in a cell by providing sufficient substrates. Thereby, the activity of NOS was detected. After coculture with HaCaT cells, the *T. rubrum* suspension was incubated with 100 *μ*l of NOS detection buffer and 100 *μ*l of reaction solution (Beyotime Biotechnology, Haimen, China) at 37°C for 20-60 min in a CO_2_ incubator. The fluorescence signal intensity was analyzed using a flow cytometer (BD FACSCanto II) at an excitation wavelength of 495 nm and an emission wavelength of 515 nm. Based on the fluorescence signal intensity, we could detect the level of NOS activity in *T. rubrum*.

### 2.7. Determination of Asymmetric Dimethylarginine (ADMA) Levels

We divided the cultures grown in liquid medium into a *T. rubrum* group and a *T. rubrum*+HaCaT cell group. The *T. rubrum* suspension supernatant of each group was incubated with 50 *μ*l of sample and 50 *μ*l of Detection Reagent A at 37°C for 60 min in a CO_2_ incubator and washed in detergent three times. Then, we added 100 *μ*l of Detection Reagent B, incubated the samples at 37°C for 30 min, and washed the plate 5 times. Next, 90 *μ*l of 3,3′,5,5′-tetramethylbenzidine (TMB) substrate was added, and the samples were incubated at 37°C for 15-25 min. Finally, we added 50 *μ*l of stop solution and immediately measured the optical density of each well using a microplate reader (SpectraMax M5, Molecular Devices, USA) at a wavelength of 450 nm. All steps refer to the manufacturer's instructions from the enzyme-linked immunosorbent assay (ELISA) kit (Cloud-Clone Corporation, USA).

### 2.8. Determination of Fungal Keratinase Activity

Keratinase activity was measured using keratin azure (Sigma). Sample supernatants (3 ml from each group) were incubated with keratin azure (10 mg) at 37°C for 72 h in 2 ml buffer (0.555 g CaCl_2_ in 50 ml of pH = 8.0 Tris-HCl buffer). Keratinase activity was determined by measuring the optical density at a wavelength of 595 nm.

### 2.9. Scanning Electron Microscopy (SEM) Observation of Morphological Changes in *T. rubrum*

Samples were fixed with 2.5% glutaraldehyde for 2 h at room temperature and stored at 4°C. Samples were sent to an electron microscope chamber for air-drying and gold spraying. Then, they were observed and imaged with a Hitachi SU8010 scanning electron microscope (Japan).

### 2.10. Transmission Electron Microscopy (TEM) Observation of Morphological Changes in *T. rubrum*

Samples were fixed with 2.5% glutaraldehyde for 2-4 h at 4°C and stored at 4°C. Samples were washed three times in PBS, postfixed for 2 h in 1% osmium tetroxide, dehydrated in ethanol followed by acetone, and embedded. Samples were cut into 60-80 nm ultrathin sections using a Leica UC7 ultramicrotome and contrasted with uranyl acetate and lead citrate. TEM studies were performed using a Hitachi HT7700 transmission electron microscope (Japan).

### 2.11. Data Analysis

All data are expressed as means ± SDs. Statistical analyses were performed using a paired-samples *t*-test and one-way analysis of variance (ANOVA), followed by post hoc analysis using the least significant difference (LSD) test or Dunnett's T3 test. A *P* value of <0.05 was considered statistically significant.

## 3. Results

### 3.1. Establishing the Optimal Conditions for HaCaT Cell Activity against *T. rubrum*

**Figure 1:**
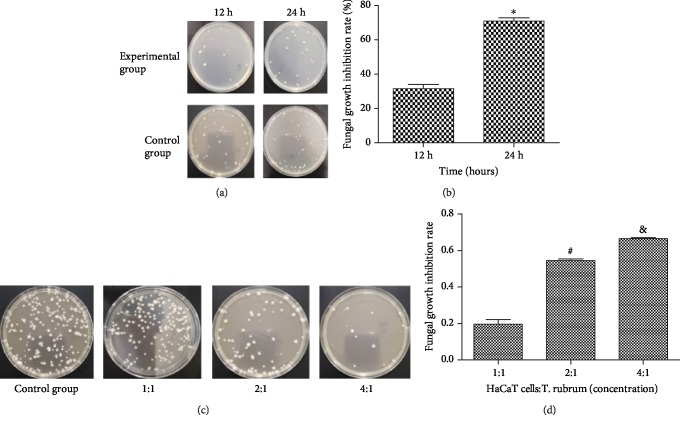
Growth inhibition rate of *T. rubrum* at different time points and for different doses of HaCaT cell intervention. (a) Incubation of the experimental group and the control group for 12 h and 24 h for assessment via the colony-counting method. (b) Growth inhibition rate of the fungus at different time points. (c) The coincubation ratios of cells : fungi were 1 : 1, 2 : 1, and 4 : 1 for assessment via the colony-counting method. (d) Growth inhibition rate of fungi treated with different coincubation ratios (^#^*P* < 0.05 vs. the 1 : 1 group, ^&^*P* < 0.05 vs. the 2 : 1 group).

Enumeration of the colony-forming units revealed that the inhibitory rates were 31.6 ± 7.0% and 70.9 ± 5.2% at 12 and 24 h, respectively. For the dose-response assay, the inhibitory rates were 19.6 ± 7.2%, 54.5 ± 2.6%, and 66.6 ± 1.5% at HaCaT cell : *T. rubrum* suspension ratios of 1 : 1, 2 : 1, and 4 : 1, respectively. Therefore, an HaCaT cell : *T. rubrum* suspension ratio of 4 : 1 and a coculture time of 24 h were used in the subsequent experiments (Figure 1).

### 3.2. Effect of NADPH Oxidase (Nox) on *T. rubrum* Activity after HaCaT Cell Intervention

**Figure 2:**
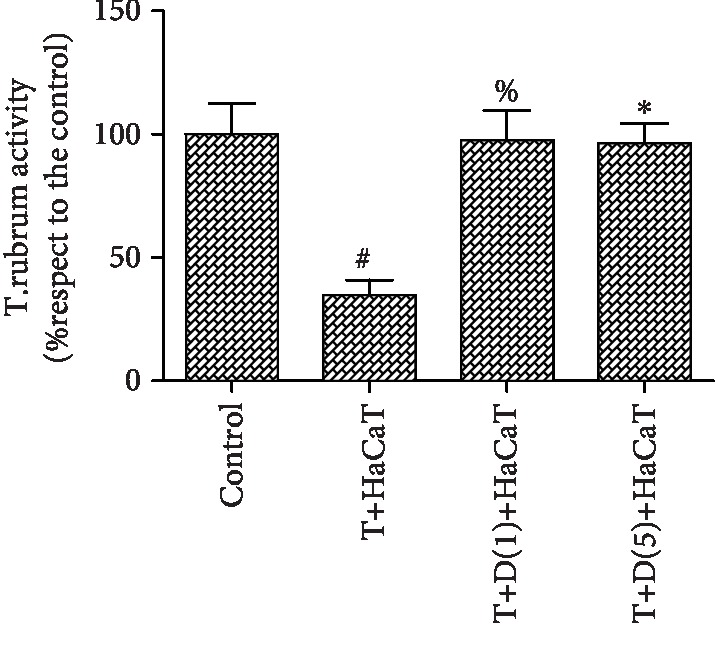
Effect of a Nox inhibitor (DPI) on *T. rubrum* activity after HaCaT cell intervention (^#^*P* < 0.05 vs. the control group, ^%^*P*, ^∗^*P* < 0.05 vs. the T+HaCaT cell group). The T+HaCaT group means that the HaCaT cells were cocultured with *T. rubrum*. The T+D(1)+HaCaT group is the *T. rubrum*+DPI(1.0 *μ*M)+HaCaT cell group, and the T+D(5)+HaCaT group is the *T. rubrum*+DPI(5.0 *μ*M)+HaCaT cell group.

After the addition of the Nox inhibitor DPI at concentrations of 1.0 and 5.0 *μ*M, fungal viability was unaffected. The inhibitor-treated groups were incubated with different concentrations of DPI (1.0 and 5.0 *μ*M) with further HaCaT cell intervention. The fungal viability with inhibitor treatment was significantly higher than that with HaCaT cell intervention alone (*P* < 0.05). However, the differences between the groups were not significant. Therefore, a DPI concentration of 1.0 *μ*M was selected for the following experiment (Figure 2).

### 3.3. Effect of NOS on *T. rubrum* Activity after HaCaT Cell Intervention

**Figure 3:**
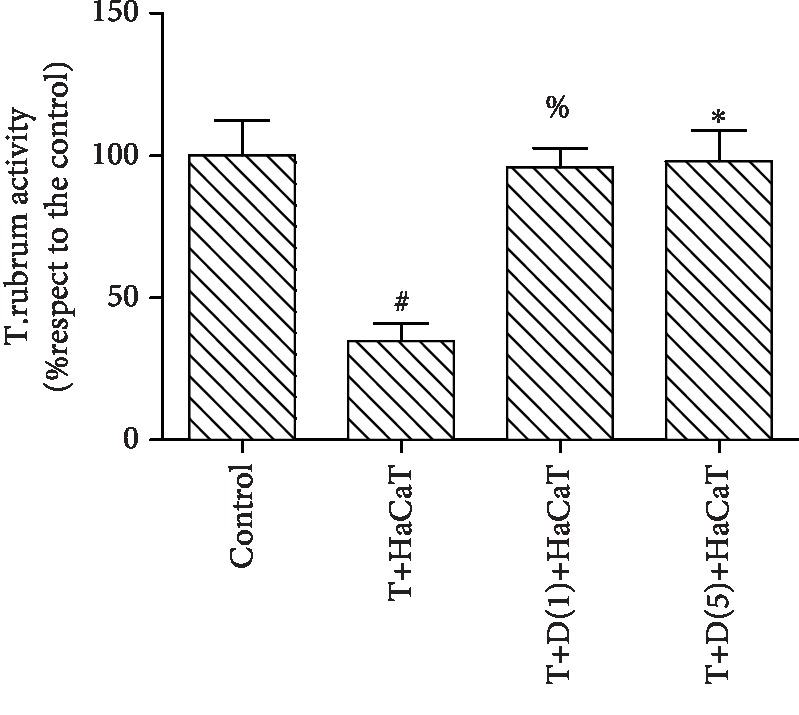
Effect of a NOS inhibitor (L-NMMA) on *T. rubrum* activity after HaCaT cell intervention (^#^*P* < 0.05 vs. the control group, ^%^*P*, ^∗^*P* < 0.05 vs. the T+HaCaT cell group). The T+HaCaT group means that the HaCaT cells were cocultured with *T. rubrum*. The T+L(0.4)+HaCaT group is the *T. rubrum*+L-NMMA(0.4 mM)+HaCaT cell group, and the T+L(0.8)+HaCaT group is the *T. rubrum*+L-NMMA(0.8 mM)+HaCaT cell group.

After the addition of the NOS inhibitor L-NMMA at concentrations of 0.4 and 0.8 mM, fungal viability was unaffected. The inhibitor-treated groups were incubated with different concentrations of L-NMMA (0.4 and 0.8 mM) with further HaCaT cell intervention. The fungal viability with inhibitor treatment was significantly higher than that with HaCaT cell intervention alone (*P* < 0.05). However, the differences between the groups were not statistically significant. Therefore, an L-NMMA concentration of 0.4 mM concentration was selected for the subsequent experiment (Figure 3).

### 3.4. HaCaT Cells Induce ROS Generation in *T. rubrum*

**Figure 4:**
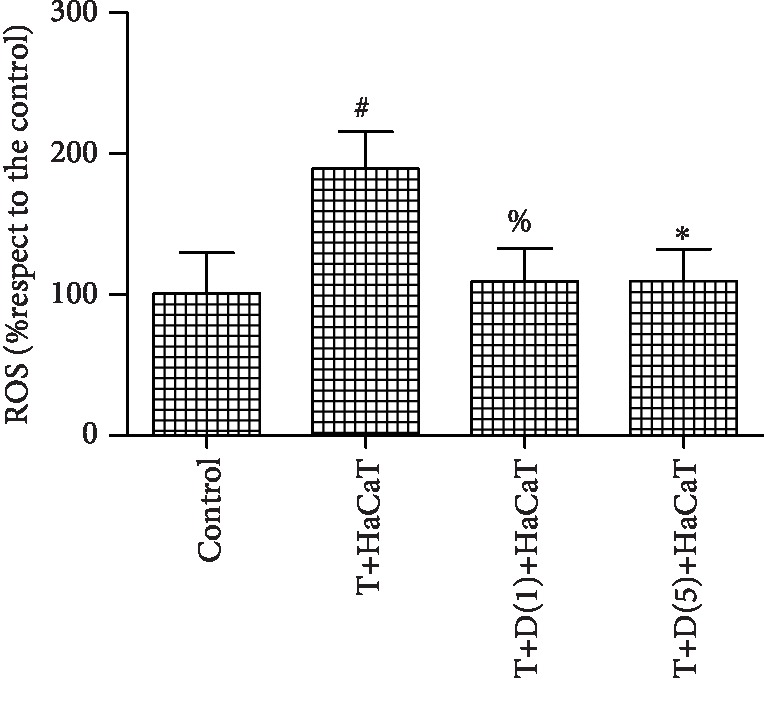
Changes in intracellular ROS levels in *T. rubrum* (^#^*P* < 0.05 vs. the control group, ^%^*P*, ^∗^*P* < 0.05 vs. the *T. rubrum*+HaCaT cell group). The T+HaCaT group means that the HaCaT cells were cocultured with *T. rubrum*. The T+D(1)+HaCaT group is the *T. rubrum*+DPI(1.0 *μ*M)+HaCaT cell group, and the T+D(5)+HaCaT group is the *T. rubrum*+DPI(5.0 *μ*M)+HaCaT cell group.

The fluorescence intensity was significantly higher (*P* < 0.05) in the *T. rubrum*+HaCaT cell group than in the control group. However, the *T. rubrum*+DPI+HaCaT cell group exhibited markedly less ROS generation than the *T. rubrum*+HaCaT cell group. In addition, the difference between the groups treated with 1 mM and 5 mM DPI was not statistically significant (Figure 4).

### 3.5. HaCaT Cells Induce NO Generation in *T. rubrum*

**Figure 5:**
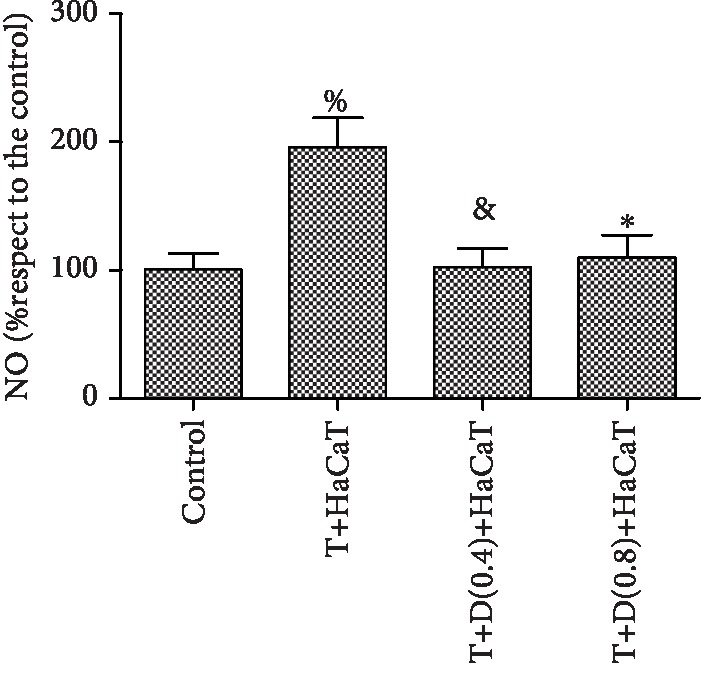
Changes in intracellular NO levels in *T. rubrum* (^%^*P* < 0.05 vs. the control group, ^&^*P*, ^∗^*P* < 0.05 vs. the *T. rubrum*+HaCaT cell group). The T+HaCaT group means that the HaCaT cells were cocultured with *T. rubrum*. The T+L(0.4)+HaCaT group is the *T. rubrum*+L-NMMA(0.4 mM)+HaCaT cell group, and the T+L(0.8)+HaCaT group is the *T. rubrum*+L-NMMA(0.8 mM)+HaCaT cell group.

Compared to the control group, the *T. rubrum*+HaCaT cell group showed a significant increase (*P* < 0.05) in the levels of NO. However, compared to the *T. rubrum*+HaCaT cell group, the *T. rubrum*+L-NMMA+HaCaT cell group exhibited markedly reduced NO generation (*P* < 0.05). Moreover, the difference between the groups treated with 0.4 mM and 0.8 mM L-NMMA was not statistically significant (Figure 5).

### 3.6. NOS Enzymatic Activity in *T. rubrum*

**Figure 6:**
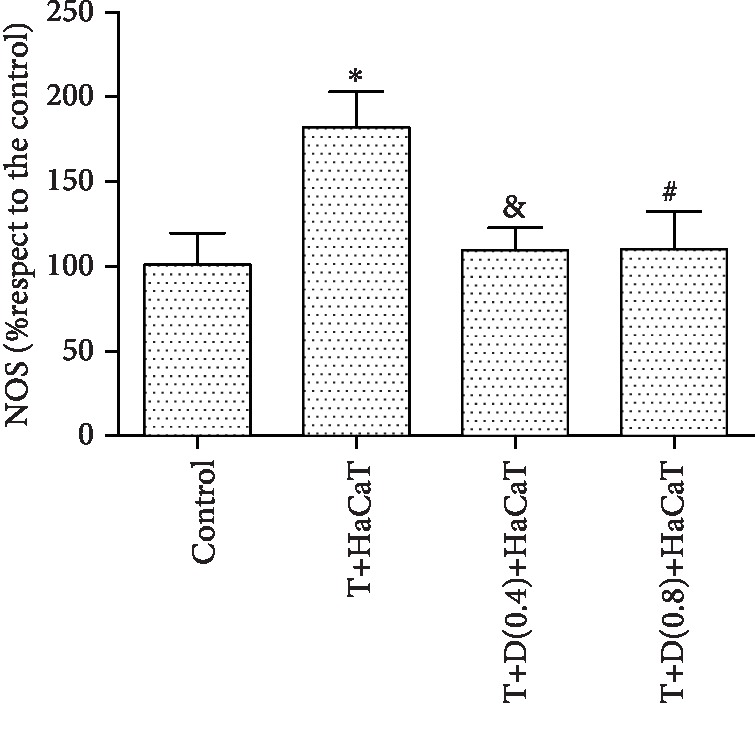
NOS activity in *T. rubrum* (^∗^*P* < 0.05 vs. the control group, ^&^*P*, ^#^*P* < 0.05 vs. the *T. rubrum*+HaCaT cell group). The T+HaCaT group means that the HaCaT cells were cocultured with *T. rubrum*. The T+L(0.4)+HaCaT group is the *T. rubrum*+L-NMMA(0.4 mM)+HaCaT cell group, and the T+L(0.8)+HaCaT group is the *T. rubrum*+L-NMMA(0.8 mM)+HaCaT cell group.

Our results indicated that the enzymatic activity of NOS in the *T. rubrum*+HaCaT cell group was significantly upregulated compared with that in the control group (*P* < 0.05). However, the *T. rubrum*+L-NMMA+HaCaT cell group exhibited markedly reduced NOS enzymatic activity compared with the *T. rubrum*+HaCaT cell group. Furthermore, the difference between the groups treated with 0.4 mM and 0.8 mM L-NMMA was not statistically significant (Figure 6).

### 3.7. ADMA Levels in *T. rubrum*

**Figure 7:**
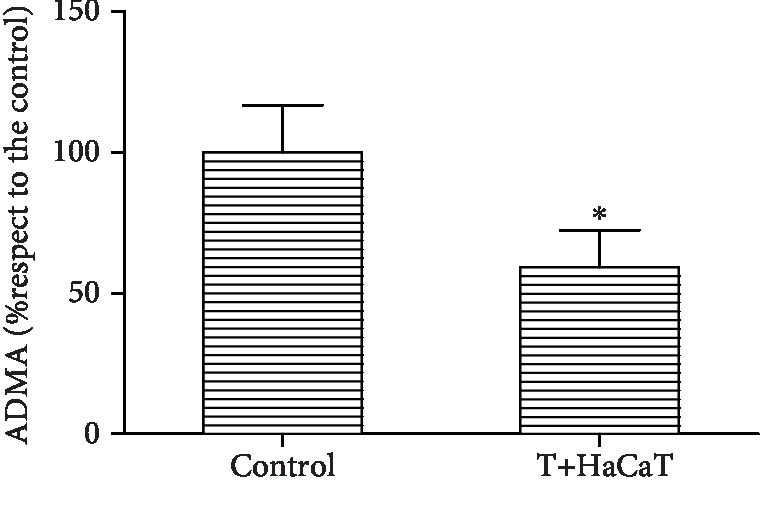
Changes in ADMA expression in *T. rubrum* (^∗^*P* < 0.05 vs. the control group). The T+HaCaT group means that the HaCaT cells were cocultured with *T. rubrum*.

The level of ADMA in the *T. rubrum*+HaCaT cell group was significantly decreased compared with that in the *T. rubrum* group (*P* < 0.05) (Figure 7).

### 3.8. Changes in Fungal Keratinase Activity

**Figure 8:**
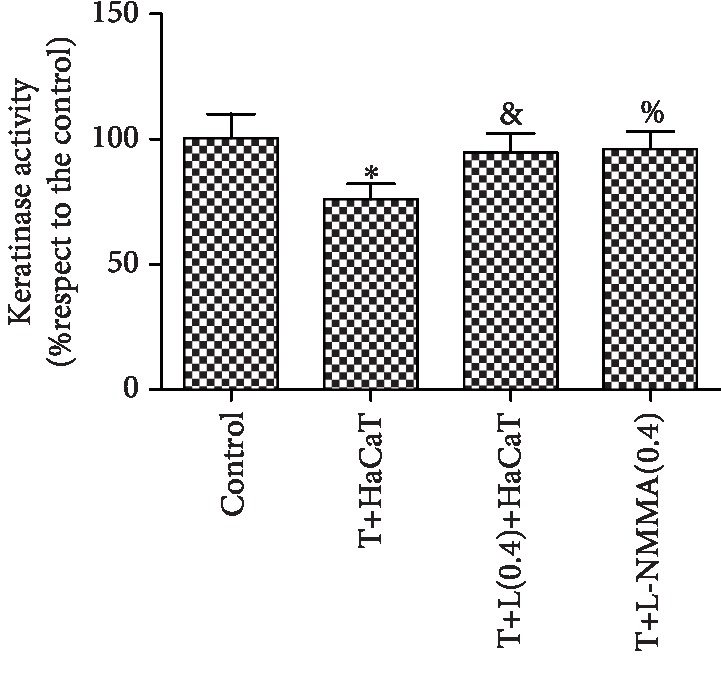
Fungal keratinase activity in *T. rubrum* (^∗^*P* < 0.05 vs. the control group, ^&^*P*, ^%^*P* < 0.05 vs. the *T. rubrum*+HaCaT cell group). The T+HaCaT group means that the HaCaT cells were cocultured with *T. rubrum*. The T+L(0.4)+HaCaT group is the *T. rubrum*+L-NMMA(0.4 mM)+HaCaT cell group, and the T+L-NMMA(0.4) group is the *T. rubrum* pretreated with 0.4 mM L-NMMA group.

**Figure 9:**
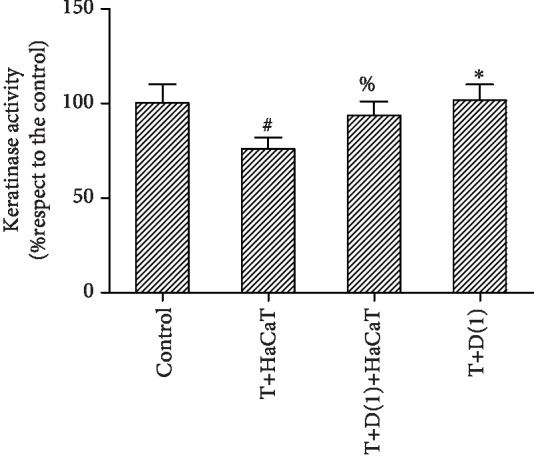
Fungal keratinase activity in *T. rubrum* (^#^*P* < 0.05 vs. the control group, ^%^*P*, ^∗^*P* < 0.05 vs. the *T. rubrum*+HaCaT cell group). The T+HaCaT group is the HaCaT cells cocultured with *T. rubrum*; the T+D(1)+HaCaT group is the *T. rubrum*+DPI(1.0 *μ*M)+HaCaT cell group; and the T+D(1) group is the *T. rubrum* pretreated with 1.0 *μ*M DPI group.

Keratinase activity decreased relative to that in the control group after coculture with HaCaT cells but increased with DPI and L-NMMA pretreatment. In addition, the difference between the activity in the group treated with DPI and L-NMMA alone and that in the control group was not statistically significant. This suggests that DPI and L-NMMA are not toxic to the fungus and do not affect the normal growth of the fungus (Figures 8 and 9). In addition, when the fungus that was pretreated with DPI and L-NMMA was cocultured with the cells, the fungal suspension containing inhibitor was washed away. Thus, DPI and L-NMMA did not contact the HaCaT cells directly and did not produce any toxic reaction to the cells.

### 3.9. Morphological Changes in *T. rubrum* Observed by SEM

**Figure 10:**
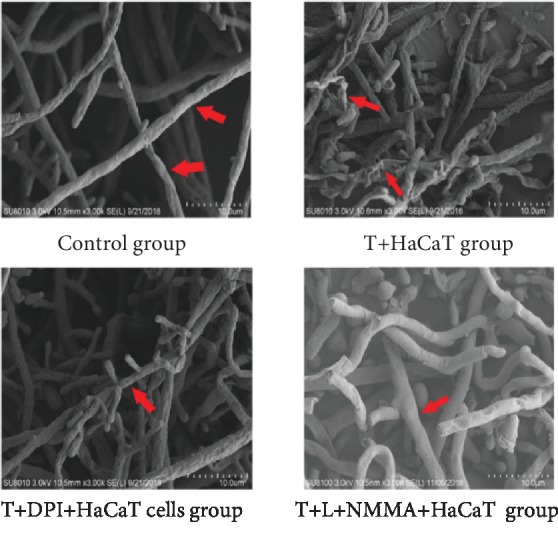
Scanning electron micrographs of *T. rubrum*+HaCaT cell intervention alone and *T. rubrum*+DPI(L-NMMA) pretreatment+HaCaT cells at 24 h (10.5 mm × 3.00 k). The T+HaCaT group means that the HaCaT cells were cocultured with *T. rubrum.* The T+DPI+HaCaT group is the *T. rubrum*+DPI(1.0 *μ*M)+HaCaT cell group, and the T+L-NMMA+HaCaT group is the *T. rubrum*+L-NMMA(0.4 mM)+HaCaT cell group. The red arrow points to the fungal morphology.

SEM observation indicated that the morphology of the hyphae and spores in the control group was normal and that the surface was smooth and plump, while the hyphae in the *T. rubrum*+HaCaT cell group were atrophic, distorted, shrunken, and irregular, and many of the hyphae were damaged and broken with surface deformation. However, compared with the *T. rubrum*+HaCaT cell group, DPI and L-NMMA pretreatment can inhibit morphological changes in the hyphae and spores, which were uniformly thick, smooth, and plump, with almost no damage. Moreover, the appearance of the fungus in the *T. rubrum*+DPI(L-NMMA)+HaCaT cell group and the fungus in the control group was not appreciably different (the red arrow points to the fungal morphology; 10.5 mm × 3.00 k) (Figure 10).

### 3.10. Morphological Changes in *T. rubrum* Observed by TEM

**Figure 11:**
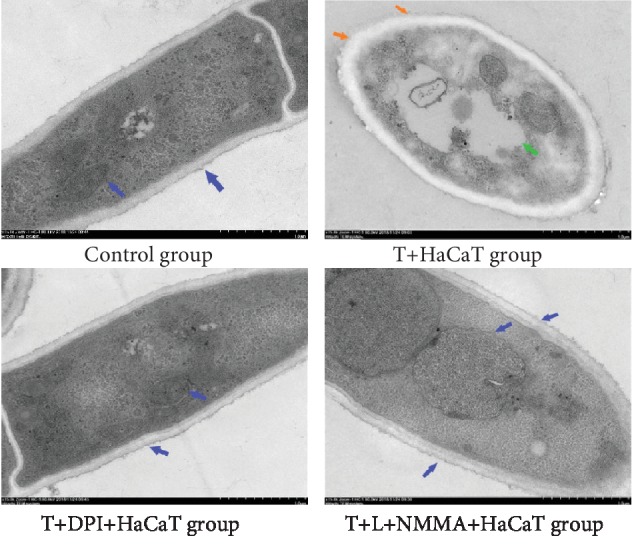
Transmission electron micrographs of *T. rubrum*+HaCaT cell intervention alone and *T. rubrum*+DPI(L-NMMA) pretreatment+HaCaT cells at 24 h (×15.0 k). The T+HaCaT group means that the HaCaT cells were cocultured with *T. rubrum*. The T+DPI+HaCaT group is the *T. rubrum*+DPI(1.0 *μ*M)+HaCaT cell group, and the T+L-NMMA+HaCaT group is the *T. rubrum*+L-NMMA(0.4 mM)+HaCaT cell group. The blue arrow points to the normal fungal internal structures, such as the cell wall, plasma membrane, and organelles. The green arrow points to vacuoles, and the orange arrow points to cell deformation, an irregular plasma membrane and a cell wall that varied in thickness at certain sites.

TEM observation demonstrated normal internal structures of the *T. rubrum* strains in the control cells. The cell wall, plasma membrane, and organelles had a normal appearance. In contrast with the control group, the *T. rubrum*+HaCaT cell group exhibited cell deformation, an irregular plasma membrane, and a cell wall that varied in thickness at certain sites. The cytoplasmic contents were degraded, as shown by the small membrane vesicles and vacuoles. However, DPI and L-NMMA pretreatment inhibited morphological changes in the yeast cells compared with those in the *T. rubrum*+HaCaT cell group; the pretreated yeast cells exhibited almost no damage. Moreover, the appearance of the fungi in the *T. rubrum*+DPI(L-NMMA)+HaCaT cell group and fungus in the control group was not appreciably different (×15.0 k) (Figure 11).

## 4. Discussion

*T. rubrum* is a common dermatophyte and one of the most important disease-causing fungi in humans [29]. In response to the binding of fungal PAMPs to particular PRRs on the keratinocyte surface, keratinocytes produce various chemokines and act both as a mechanical and an immunological barrier to pathogens [30]. In contrast, the mechanism that is active in fungi in response to keratinocytes is unclear.

In this study, HaCaT cells inhibited the activity of *T. rubrum*. SEM observation of the fungus after coculture with HaCaT cells showed that hyphae were damaged and broken with surface deformation. TEM revealed that the cytoplasm was disrupted, the organelles were destroyed, and necrotic cells were found. Moreover, after HaCaT cell intervention, the fungal keratinase activity decreased. Keratinase is considered one of the best characterized virulence factors of dermatophytes in skin infection and fundamentally important for fungal invasion and dissemination throughout the stratum corneum of the host [31]. These data are consistent with those from a study reporting that several types of cells, including keratinocytes, fibroblasts, T lymphocytes, and neutrophils, are involved in epithelial protection against fungal invasion [32, 33]. Our results indicated that keratinocytes can inhibit the growth and pathogenicity of *T. rubrum*.

ROS and NO have been reported to play key roles in the cellular activity of fungi [34]. In this study, after HaCaT cell intervention, the intracellular ROS levels in the fungus were consistent with the degree of fungal damage and were higher than those in the control group. Nathan and Shiloh reported that when exposed to external toxins and stimulation, ROS production in cells immediately increased [35]. Due to their rapid and extensive production, small molecular size, and transmembrane diffusibility, ROS play a crucial role in antifungal activity [36]. Combined with the results of our study, these results show that ROS may play an important role in the antifungal effects of HaCaT cells against *T. rubrum*.

The most important enzymatic ROS generating system is the NADPH oxidase (Nox) family [37]. Nox produces ROS by transferring electrons from NADPH to molecular oxygen to produce superoxide and other ROS and is ubiquitous in filamentous fungi [38]. Rui et al. reported that the exogenous addition of the Nox inhibitor DPI can reduce cytosolic ROS accumulation in *Ganoderma lucidum*, an edible fungus [39]. To investigate whether Nox is involved in the antifungal effects of HaCaT cells on *T. rubrum*, we added the inhibitor DPI to a fungal suspension for 2 h of pretreatment before HaCaT cell intervention. The fungal viability was significantly higher than that observed for coculture with HaCaT cell alone. SEM observation indicated that the hyphae pretreated with DPI exhibited almost no damage. TEM observation of these hyphae indicated a regular morphology with intact cell walls and cytoplasm. The levels of intracellular ROS were also significantly lower in the *T. rubrum*+DPI+HaCaT cell group than in the *T. rubrum*+HaCaT cell group. Thus, DPI reduced the antifungal effects on *T. rubrum* from oxidative damage induced by HaCaT cells. In addition, *T. rubrum* pretreated with DPI showed higher keratinase activity than *T. rubrum* cocultured with HaCaT cells alone. We thus hypothesized that DPI inhibits Nox expression and reduces HaCaT cell-induced ROS accumulation to weaken the antifungal effects of HaCaT cells and increase keratinase activity.

ROS can also react with oxides of nitrogen, such as NO, to generate damaging reactive nitrogen species (RNS), such as peroxynitrite [15]. In this study, the levels of intracellular NO and NOS in fungi cocultured with HaCaT cells were consistent with the degree of fungal damage and were higher than those in the control group. The synthesis of NO in eukaryotic cells occurs via the oxidation of L-arginine by NOS [18]. NO, which is a free radical but not highly reactive, can react with superoxide anions to form more reactive products, such as ONOO and NO [40]. Spater et al. [41] reported that *Malassezia* spp. can generate RNS in a medium in vitro. Furthermore, studies have clearly demonstrated the production of NO and ONOO in the fungal cell; NO and ONOO appear to be critical for killing *T. rubrum* [12]. NO was reported to inhibit the mycelial growth, sporulation, and germination of postharvest fungi [42]. Moreover, Tümer et al. found that NO exerts direct fungistatic activity via DNA damage, lipid peroxidation, and enzyme inactivation [24]. Combined with the results of our study, these results show that NO may play an important role in the antifungal effects of HaCaT cells against *T. rubrum*.

NO is produced by the conversion of L-arginine into L-citrulline through a reaction catalyzed by NOS. Although L-arginine is the main precursor to NO, the regulation of NO production is multifactorial. NO production is not limited by circulating L-arginine but instead is regulated by NOS activation and NOS inhibition, such as that produced by L-NMMA [43]. In addition, the NOS inhibitor L-NMMA has been reported to decrease the content of NO as well as the level of NOS. To investigate whether NOS is involved in the antifungal effects of HaCaT cells on *T. rubrum*, we added the inhibitor L-NMMA to a fungal suspension for 2 h before HaCaT cell intervention. The fungal viability was significantly higher than that observed for coculture with HaCaT cells alone. SEM observation indicated that hyphae pretreated with L-NMMA displayed normal morphology and appeared almost identical to those in the control group. TEM observation of these hyphae indicated a regular morphology with regular cell walls, cytoplasm, and cellular organelles. The levels of intracellular NO and NOS activity were also significantly decreased. In addition, fungus pretreated with L-NMMA showed higher keratinase activity than *T. rubrum* cocultured with HaCaT cells alone. We thus hypothesized that L-NMMA inhibits NOS expression, reduces HaCaT cell-induced NO accumulation, and increases keratinase activity.

ADMA functions as a competitive inhibitor of NOS and thus leads to reduced NO production [44]. The expression of ADMA in yeast proteins has been demonstrated. *Saccharomyces cerevisiae* cells in log-phase growth exhibited 11-fold higher ADMA expression than cells of this yeast under heat shock or in a stationary phase [45]. To study whether ADMA is also involved in the inhibition of *T. rubrum* growth by HaCaT cells, we cocultured HaCaT cells with *T. rubrum*, not only to assess NOS expression and the NO level but also to measure the ADMA level. We found that after intervention with HaCaT cells, the activity of *T. rubrum* was decreased, accompanied by a decline in the ADMA concentration and increases in NOS expression and NO levels. We thus hypothesized that HaCaT cells can increase the expression of NOS by decreasing the level of ADMA, resulting in an increase in the synthesis of NO which causes nitrosative damage in fungi; thus, the growth of *T. rubrum* is inhibited. We believe that this mechanism is one of the important mechanisms by which HaCaT cells inhibit fungal growth. However, the mechanism by which HaCaT cells affect the ADMA level needs further study.

In summary, our study showed that HaCaT cells can inhibit *T. rubrum* growth and fungal pathogenicity in vitro. Although the mechanism underlying this effect is not entirely clear, the production of large amounts of ROS and NO, inducing a cascade of reactions, may play an important role. Our results prompted us to hypothesize that this cascade of reactions is caused by an increase in the levels of Nox and NOS as well as a reduction in the level of ADMA, which may be the targets in *T. rubrum*. We inferred from the above research that because of the immunocompetence of keratinocytes and other cells in the skin, when only a few fungi invade the skin, we can treat fungal infections with therapies that repair the skin barrier instead of overusing antibiotics and other interventions. These results provide novel insight regarding the mechanisms underlying the responses of *T. rubrum* to HaCaT cells and establish potential therapeutic approaches and interventional targets. However, further in vivo studies will be required for confirmation.

## Data Availability

The data used to support the findings of this study are available from the corresponding author upon request.
